# Cardiovascular-related proteomic changes in ECFCs exposed to the serum of COVID-19 patients

**DOI:** 10.7150/ijbs.78864

**Published:** 2023-03-05

**Authors:** Lucía Beltrán-Camacho, Santosh D. Bhosale, Daniel Sánchez-Morillo, Ismael Sánchez-Gomar, Marta Rojas-Torres, Sara Eslava-Alcón, Mario Martínez-Torija, Mª Angeles Ruiz de Infante, Mª Dolores Nieto-Martín, Manuel A. Rodríguez-Iglesias, Juan A. Moreno, Esther Berrocoso, Martin R. Larsen, Rafael Moreno-Luna, Mª Carmen Durán-Ruiz

**Affiliations:** 1 Biomedicine, Biotechnology and Public Health Department, University of Cadiz, 11002 Cadiz, Spain; 2 Biomedical Research and Innovation Institute of Cadiz (INiBICA), 11002 Cadiz, Spain; 3 Department of Biochemistry and Molecular Biology, University of Southern Denmark, 5230 Odense, Denmark; 4 Automation Engineering, Electronics and Computer Architecture and Networks Department, University of Cadiz, 11009 Cadiz, Spain; 5 Department of Nursing, National Paraplegic Hospital, Toledo, Spain; 6 Occupational Health Service, National Paraplegic Hospital, SESCAM, 45071, Toledo, Spain; 7 Internal Medicine Department, University Hospital Virgen del Rocío, Seville, Spain; 8 UGC Microbiología, University Hospital Puerta del Mar, Avda. Ana de Viya 21, 11009, Cádiz, Spain; 9 Cell Biology, Physiology and Immunology Department, Agrifood Campus of International Excellence (ceiA3), University of Cordoba, 14014, Córdoba, Spain; 10 Maimonides Biomedical Research Institute of Cordoba (IMIBIC), UGC Nephrology, Hospital Universitario Reina Sofia, 14004, Cordoba, Spain; 11 Psychology Department, University of Cádiz, 11510, Puerto Real, Spain; 12 Biomedical Research Networking Center for Mental Health Network (CIBERSAM), Institute of Health Carlos III, 28029, Madrid, Spain; 13 Laboratory of Neuroinflammation, National Paraplegic Hospital, 45071 Toledo, Spain; 14 Redes de investigación cooperativa orientadas a resultados en salud, Enfermedades vasculares cerebrales, RICORS-ICTUS, SESCAM

**Keywords:** COVID-19, ECFCs, Viral infection, RNA metabolism, cardiovascular diseases, endothelial dysfunction, autophagy, proteomics, mass spectrometry

## Abstract

Severe acute respiratory syndrome coronavirus 2 (SARS-CoV-2) infection significantly affects the cardiovascular system, causing vascular damage and thromboembolic events in critical patients. Endothelial dysfunction represents one of the first steps in response to COVID-19 that might lead to cardiovascular complications and long-term sequelae. However, despite the enormous efforts in the last two years, the molecular mechanisms involved in such processes remain poorly understood. Herein, we analyzed the protein changes taking place in endothelial colony forming cells (ECFCs) after the incubation with the serum from individuals infected with COVID-19, whether asymptomatic or critical patients, by application of a label free-quantitative proteomics approach. Specifically, ECFCs from healthy individuals were incubated *ex-vivo* with the serum of either COVID-19 negative donors (PCR-/IgG-, n:8), COVID-19 asymptomatic donors at different infective stages (PCR+/ IgG-, n:8and PCR-/IgG+, n:8), or hospitalized critical COVID-19 patients (n:8), followed by proteomics analysis. In total, 590 proteins were differentially expressed in ECFCs in response to all infected serums. Predictive analysis highlighted several proteins like CAPN5, SURF4, LAMP2 or MT-ND1, as highly discriminating features between the groups compared. Protein changes correlated with viral infection, RNA metabolism or autophagy, among others. Remarkably, the angiogenic potential of ECFCs in response to the infected serums was impaired, and many of the protein alterations in response to the serum of critical patients were associated with cardiovascular-related pathologies.

## Background

Coronavirus disease 2019 (COVID-19) was declared as a global pandemic on March 11, 2020, because of the severe acute respiratory syndrome coronavirus 2 (SARS-CoV-2) infection, provoking more than 6,7 million deaths worldwide (www.covid19.who.int). SARS-CoV-2 not only affects the respiratory system, it also provokes severe vascular damage as well as thromboembolic events responsible for many associated clinical complications 1, 2. Importantly, in relation to the former processes, endothelial dysfunction plays a significant role in the pathogenesis of COVID-19, either by direct infection through SARS-CoV-2 3 or as result of the activation of inflammatory leukocytes promoted by the vascular endothelium, leading to a cytokine storm responsible for systemic inflammation 4-6.

Although the effects are more potentiated in severe patients, endothelial dysfunction takes place even in asymptomatic individuals 7, and different signs of persistent endothelial activation and related inflammation can be found even in convalescent COVID-19 patients, including elevated levels of circulating endothelial cells (CECs) 8, 9. Hence, a better understanding of the initial stages in which SARS-CoV-2 affects the endothelium is required, in order to predict or prevent unwanted secondary effects, and the risk of suffering from long-term cardiovascular (CV) complications 5, 7.

To date, different approaches have arisen to evaluate the potential mechanisms by which SARS-CoV-2 might promote endothelial damage, from *in vitro* studies with human organoids and organ-on-chip platforms 4, 10-12, to *in vivo* assays with animals expressing ACE2 and further infected with SARS-CoV-2 13. Very recently, we described a cell model to analyze the response of vascular cells to SARS-CoV-2 infection, based on the incubation of circulating angiogenic cells (CACs), also considered as CECs, with the serum of COVID-19 asymptomatic donors, identifying many proteins related to endothelial dysfunction and inflammatory response after viral infection, corroborating the potential of this approach 7. In the current study, we have extended our strategy by evaluating the effect of the serum factors from asymptomatic to critical COVID-19 patients over endothelial colony forming cells (ECFCs) isolated from white adipose tissue of healthy adults, by application of advanced mass spectrometry (MS)-based proteomics methods. We and other researchers have already shown that these cells present a robust clonogenic and proliferative potential and they are known to promote vascular repair 14-18. On the other hand, under pathological environments, ECFCs become dysfunctional; moreover, in response to adverse conditions, impaired ECFCs might contribute to the endothelial dysfunction related to cardiovascular diseases (CVDs) 19-22. Remarkably, elevated levels of ECFCs have been found in 3 months post-COVID patients 9. Thus, ECFCs may not only provide novel opportunities in identifying biomarker of post-COVID endothelial damage, but also represent an optimal candidate to tackle SARS-CoV-2 endothelial infection and a platform to evaluate therapeutic strategies against the disease.

## Methods

### Serum sample acquisition

**Figure 1:**
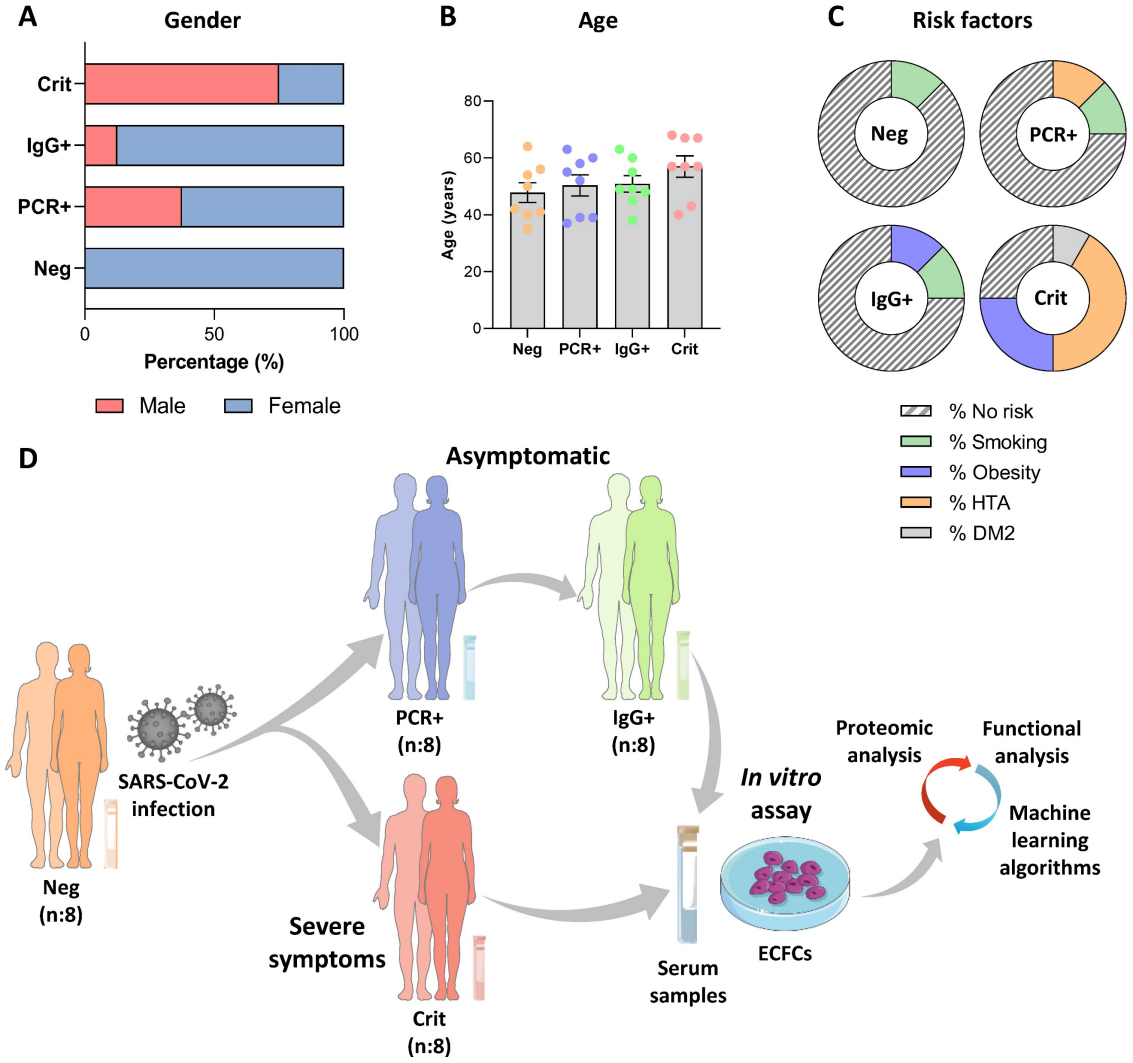
** Study population characteristics and schematic representation of the experimental assay.** Graphical representation of the donors' **A)** gender, **B)** age and **C)** risks factors for each group. **D)** Schematic representation of the experimental assay. The serums from COVID-19 patients in different stages, including negative, asymptomatic and critical individuals were collected: SARS-CoV-2 negative (PCR-/IgG-, n:8) and SARS-CoV-2 positive, at the peak of infection (PCR+/IgG-), asymptomatic (n:8) and critical (n:8), or after the infective peak (PCR -/IgG +, n:8). Next, ECFCs from healthy donors were incubated with all four sets of serum samples and a label free quantitative approach was performed, followed by bioinformatics analysis (statistic and functional classification).

The study was conducted with COVID-19 negative donors and asymptomatic individuals (PCR+/IgG- and PCR-/IgG+) recruited at the National Paraplegic Hospital (SESCAM), Toledo, Spain during April-May 2020 and critical COVID-19 patients admitted at the COVID-19 hospital, Seville, Spain, and the Puerta del Mar University Hospital, Cádiz, Spain, during May-June 2021. Donor characteristics are shown in Figure 1A-C. A SARS-CoV-2 qPCR analysis from nasopharyngeal samples and an ELISA assay testing for specific IgG and IgM antibodies (IME00136 and IME00137; Erba Mannheim) were performed to determine their status. Briefly, peripheral blood samples were collected using serum separator tubes (SSTTM II advance, BD Vacutainer^®^), centrifuged and stored at -80 ºC, as described 7.

Donors were classified in four different groups: COVID-19 negative donors (Neg, n:8), with both qPCR and antibody's test (PCR-/IgG-), asymptomatic patients PCR positive (PCR+, n:8) or IgG positive (IgG+, n:8) at the time of blood collection, and critical COVID-19 patients that required hospitalization (Crit, n:8) (Figure 1D).

### ECFCs isolation and culture

ECFCs were isolated from normal subcutaneous white adipose tissue and cultured as previously described 18. ECFCs were purified by magnetic activated cell sorting using CD31-coated magnetic beads, plated in 1% gelatin coated plates and incubated in EBM-2 media plus 20% FBS and Single Quots growth factors (except for hydrocortisone) (Lonza). ECFCs were characterized *in vitro* and *in vivo* as described 18.

### ECFCs incubation *ex vivo* with patients' serum

ECFCs were washed several times with PBS 1X, to discard any remaining traces of FBS from the initial conditioned media, and then incubated for 24 h (37 ºC, 5% CO_2_) with EBM-2 medium containing 10% serum of the Neg, PCR+, IgG+ and Crit groups (n:8 per group), as described 7. Cells were collected using Trypsin-EDTA 1X (X0930-100; Biowest), centrifuged and washed once with PBS 1X.

### Proteomic analysis

The proteome changes of ECFCs in response to the incubation with the different sets of serum samples (Neg n:8; PCR+ n:8; IgG+ n:8; Crit n:8) were analyzed using tandem mass spectrometry (LC-MS/MS)-based label free quantitative (LFQ) analysis. Briefly, proteins were extracted by resuspending ECFCs pellets in 8M urea containing protease inhibitors (04693132001; Roche) and their amount was estimated using Qubit Fluorometric system (ThermoFisher Scientific) following manufacturer´s guidelines. Next, 50 µg of proteins in 8M urea per sample were reduced (10 mM Dithiothreitol) and alkylated (50 mM Iodoacetamide), and then diluted four times with 50 mM ammonium bicarbonate prior to digestion with Trypsin/LysC (V5073; Promega) (enzyme/substrate ratio 1:50), at 37 °C, overnight. Finally, digestion was quenched with 0.1% TFA before peptide purification with C18 micro-columns, as described 23, and eluates were dried with a speed-vac system.

Digested peptides were reconstituted in 0.1% formic acid (FA) and a NanoDrop (DeNovix, DS-11 Spectrophotometer) was used to estimate the peptide concentration. An amount equivalent to 200 ng of peptides were analyzed on a timsTOF Pro mass spectrometer (Bruker Daltonics) interfaced with Easy nLC (Thermo Scientific). Briefly, peptides were separated on an Aurora Series UHPLC emitter column (250 mm X 75 µm id, 1.6 µm C18) from IonOpticks, using the solvents A (0.1% FA) and B (95% acetonitrile with 0.1% FA). Peptides were eluted at flow rate of 300 nL/min, with increasing % of solvent B from 5 to 45% in 60 min (from 5 to 25% in 50 min followed by increasing to 45% in 10 min) and further column washing with 95% B. The column temperature was maintained to 45 ºC. The instrument was operated in diaPASEF mode via Captive nano-electrospray source (Bruker Daltonics) at 1400 V with an accumulation time of 100 ms and a ramp of 100 ms. A 25 m/z precursor isolation width was used to cover 400 to 1200 m/z, covering an ion mobility range /1/K_0_) from 0.60 to 1.60 V.s/cm^2^ 24.

### Data processing and statistics

diaPASEF files were analyzed using Spectronaut (v 15.2.210819.50606, Biognosys AG) in directDIA™ mode with default settings except protease selected were Trypsin and LysC. The detailed description of all the parameters can be found in Table S8.

The Spectronaut output was then exported into tabular format for further analysis. In the Perseus software 25, protein intensity values were log2 transformed and samples were categorically annotated to define the conditions. A t-test differential expression analysis was used with a permutation-based FDR calculation. Proteins were considered as differentially expressed between the groups (Neg, PCR+, IgG+ and Crit) when FDR < 0.05 and log_2_ foldchange > 1 (up-regulated) or < -1 (down-regulated). These changes were confirmed afterwards with GraphPad Prism 9 software. Data were presented as box plots graphs representing median, min and max value and showing all points.

Additional data processing was done using Venny v2.1^26^, Python, R and MetaboAnalyst 27. Specifically, Clustergrammer was used to generate an interactive heatmap 28. The functional role of proteins was analyzed using Ingenuity Pathway Analysis (IPA) software, Reactome (https://reactome.org/), Erichr (https://maayanlab.cloud/Enrichr/), String (https://string-db.org) and Coronascape (COVID-19 Reference Gene Lists (metascape.org).

### Machine learning algorithms

We explored the potential of machine learning techniques to classify ECFCs treated with the serum from asymptomatic (PCR+, IgG+) or COVID-19 negative individuals, and from critical COVID-19 patients. Due to the limited generalizability of the results imposed by the small size of the data set, three low complexity models were used: MLR, NB and LSVM. Feature selection was used to identify proteins with high discriminant power to reduce the high dimensionality of the data set 29 and circumvent the so-called curse of dimensionality. Attribute sets were evaluated by using a wrapper 5-fold cross validation learning scheme. Area under the ROC curve (AUC) was used to assess the performance of attribute combinations. The space of attribute subsets was bidirectionally searched by greedy hill climbing augmented with a backtracking facility. The number of consecutive non-improving nodes to allow before terminating the search was fixed at 5. The following metrics of performance for each classifier were calculated using 5-fold cross validation: accuracy, AUC, true and false positive rates, recall and Kappa statistic. WEKA data mining software 30 and MATLAB (The MathWorks Inc., Natick, USA) were used for building the models.

### *In silico* interaction analysis between serum and ECFCs altered proteins

In order to predict serum factors potentially responsible of the changes seen in ECFCs, we conducted an exhaustive literature search, selecting proteomic studies identifying proteins altered in the serum of asymptomatics or critical COVID-19 patients (Table S9). Next, an *in-silico* analysis was performed with Fluorish software (Flourish | Data Visualization & Storytelling), evaluating potential interactions between the proteins altered in the serum of COVID-19 patients' vs negative controls (detected at least in 2 of the selected articles) with the protein changes identified in ECFCs in response to the serum from COVID-19 patients.

### Angiogenesis assay

A tube formation assay was performed to evaluate the effect of the serum from COVID-19 patients over the ECFCs angiogenic potential. ECFCs (15000 cells/well) were seeded into a 96 well angiogenesis μ-plate (Ibidi, 81506) pre-coated with 10 μl matrigel (Bioscience, 356231), as described 31, and incubated for 24 h (37 ºC, 5% CO2) with EBM-2 medium containing 10% serum of the Neg, PCR+, IgG+ and Crit groups (n:4 per group), in triplicates. In addition, ECFCs were incubated with 35 ng/ml Fibroblast Growth Factor (FGF, R&D Systems) and 15 mM sulforaphane (S4441-5MG, Sigma) as angiogenic activator and inhibitor controls respectively. After 24 hours, images were taken per well with an inverted phase-contrast microscope and the number of meshes were quantified.

### Anti-inflammatory assay

ECFCs were seeded in 6-well plates, previously coated with 1% gelatin, at a density of 30000 cells/cm^2^ in EBM-2 medium and 10% FBS. After 12 h of incubation (37 ºC, 5% CO_2_), the medium was replaced, and cells were incubated with EBM-2 medium containing 10% of serum from the Neg, PCR+, IgG+ and Crit groups (n:3 per group). In addition, another set of cells were incubated with EBM-2 with 2% FBS with or without 10 ng/ml of tumor necrosis factor-α (TNF-α; R&D Systems), as negative and positive controls of the inflammatory response respectively. All wells were incubated (37 ºC, 5% CO_2_) for another 5 hours. Cells were then detached and washed with cytometry buffer (1x PBS, 2.5% FBS, 2 mM EDTA), and then incubated with anti-human VCAM-1 antibody (305809; Biolegend) for 20 min at 4ºC in the dark. Samples were analyzed using CytoFLEX cytometer (Beckam Coulter, USA) and CytExpert software. Finally, data was analyzed with FlowJo v10.4 software.

## Results

**Figure 2:**
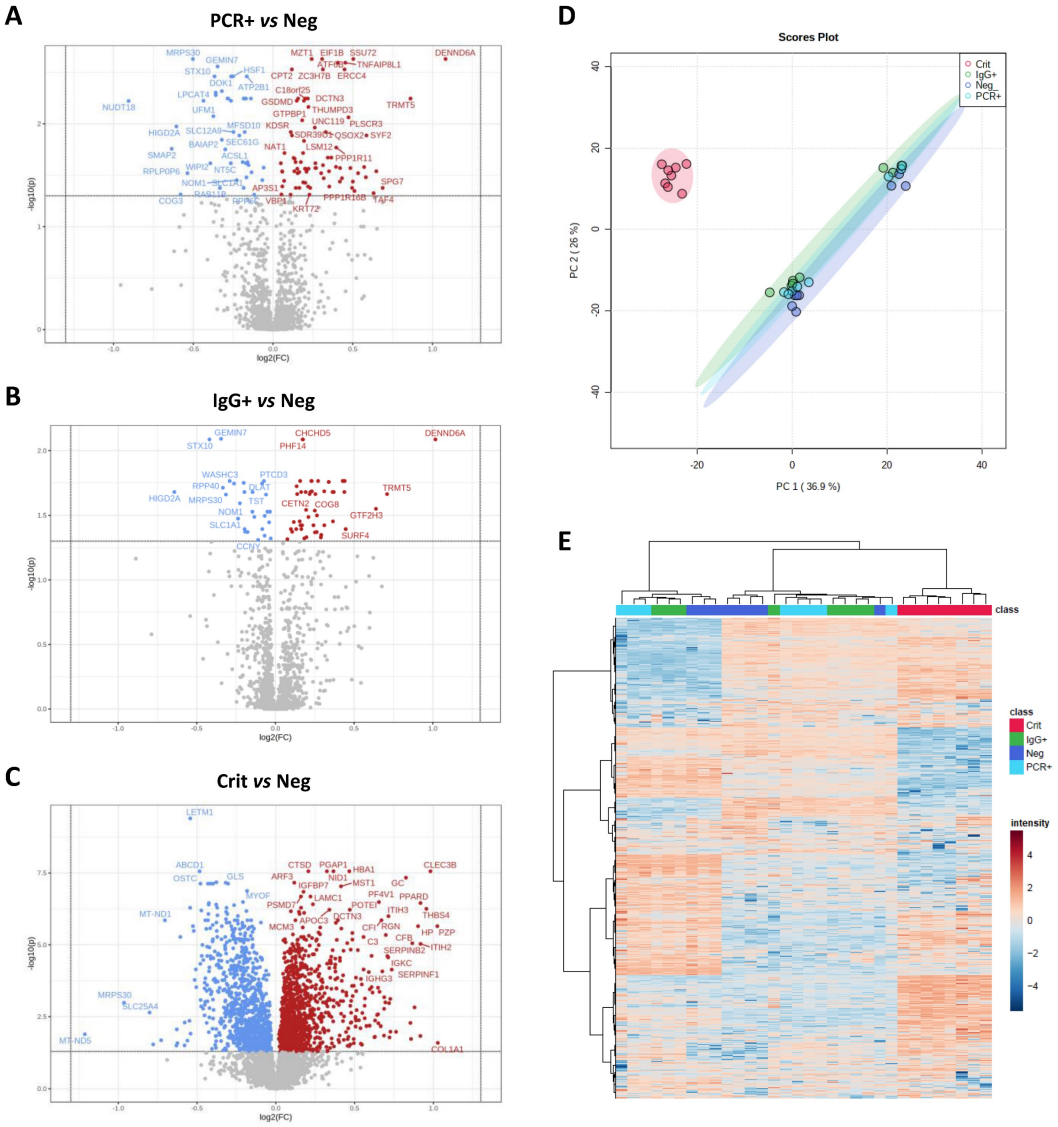
** ECFCs proteomic changes in response to the serum of COVID-19 patients in different stages and symptomatology.** Volcano plots representative of protein up (red) and down-regulated (blue) in ECFCs incubated with the serum of **A)** PCR+ *vs* Neg donors, **B)** IgG *vs* Neg and **C)** Crit *vs* Neg. **D)** Principal component analysis. **E)** Hierarchical cluster representing the differential protein profiles. Interactive heatmap available as a supplementary file in html format.

**Figure 3:**
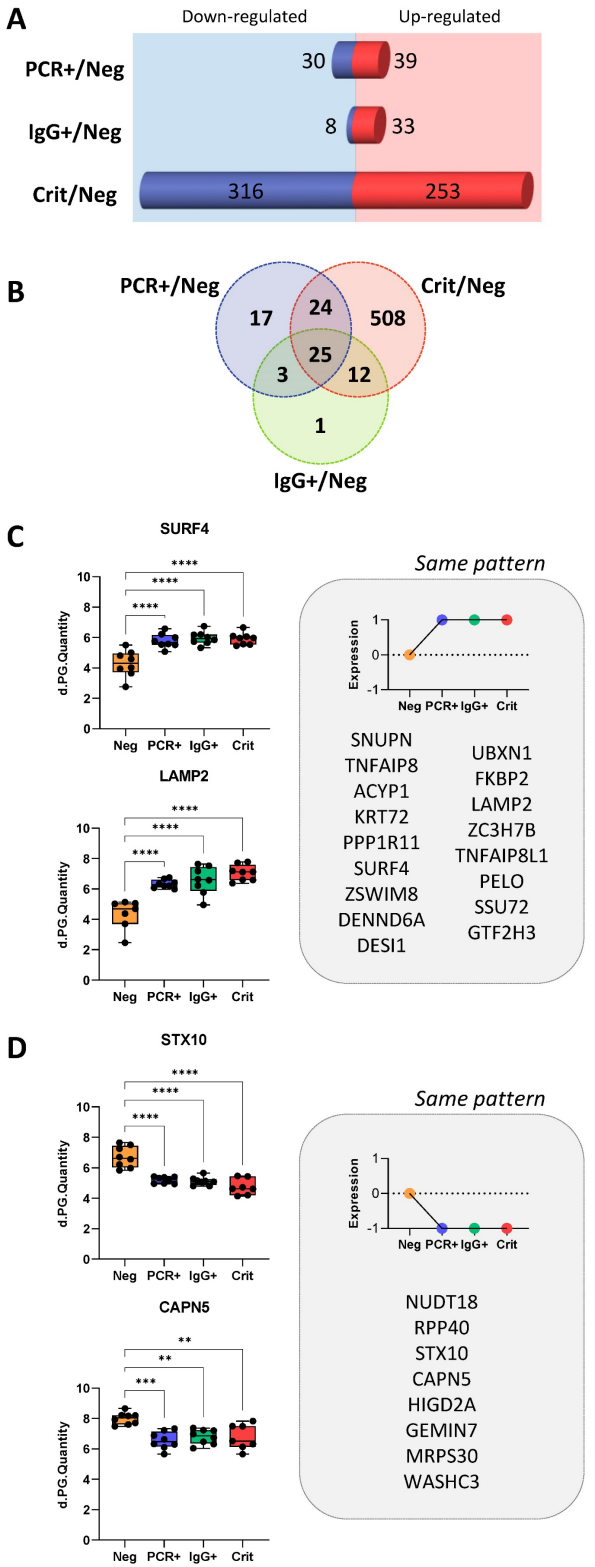
** Common protein changes in ECFCs in response to asymptomatic and critical COVID-19 serum. A)** Representation of the number of proteins up- (red) and down-regulated (blue) in ECFCs incubated with the serum of asymptomatic (PCR+, IgG+) or Crit patients compared to ECFCs + Neg. **B)** Venn's diagram showing the overlapping of proteins differently expressed between ECFCs incubated with the serum of asymptomatic (PCR+, IgG+) or Crit compared to ECFCs + Neg. From the 25 common proteins altered in ECFCs incubated with the serum of infected donors (PCR+, IgG+ and Crit) compared with ECFCs+Neg, the proteins up- **C)** or down-regulated **D)** are shown, including representative graphs with the LFQ intensities registered for some of the proteins highlighted by predictive analysis. *p-value<0.05, **p-value<0.01, ***p-value<0.001, ****p-value<0.0001.

A total of 5052 proteins (4883±129 on average) were identified in ECFCs treated with the serum from COVID-19 negative (ECFCs+Neg, n:8), PCR+ (ECFCs+PCR, n:8) and IgG+ (ECFCs+IgG, n:8) asymptomatics, and critical COVID-19 patients (ECFCs+Crit, n:8). The statistical analysis identified 590 differentially expressed proteins between these groups (Figure 2A-C). The proteome profile of ECFCs incubated with the serum of critical COVID-19 patients was clearly different than the rest of conditions, as indicated by the principal component analysis (PCA, Figure 2D) and the hierarchical clustering classification (Figure 2E). Furthermore, compared to the other groups, ECFCs+Crit reported the highest number of protein changes (up and down-regulated) (Figure 3A and B). Nevertheless, protein differences were also found in ECFCs treated with the serum of asymptomatic (PCR+ and IgG+) *vs* ECFCs+Neg control donors. Full information regarding identification and quantification data (log2 fold changes and p-values) can be found in Tables S1-4.

### Common protein changes in ECFCs in response to asymptomatic and critical COVID-19 serum

From the total of 590 differentially expressed proteins, 508 were identified only in ECFCs+Crit *vs* ECFCs+Neg cells, while 24 of them were commonly altered between ECFCs+Crit and ECFCs+PCR, and 25 proteins were commonly altered in response to the serum factors of infected individuals, COVID-19 asymptomatic (PCR+ and IgG+) donors and critical patients, compared with ECFCs+Neg (Figure 3A-D).

According to Reactome Pathway Database (Table S5), the 25 common proteins up- or downregulated in ECFCs exposed to the serum of infected individuals, were mostly related to the metabolism of RNA (RPP40, GTF2H3, SNUPN, GEMIN7) and also non-coding RNA (SNUPN, GEMIN7), including RNA polymerase activity and regulation (GTF2H3), intra-Golgi and Golgi-to-ER trafficking (SURF4, STX10), as well as chaperone mediated autophagy (LAMP2). In addition, the analysis revealed that some of these proteins had been previously associated to SARS-CoV-2 early (FKBP2; UBXN1; PPP1R11), middle (UBXN1; PPP1R11; CAPN5) and late-stage infection in human male blood (FKBP2; UBXN1; PPP1R11; SURF4; SSU72).

Therefore, these protein changes seem relevant since they remained even when ECFCs were exposed to the serum of individuals that had overcome the infection with no apparent symptoms (ECFCs+IgG).

### Differential protein expression patterns in ECFCs exposed to asymptomatic serums

**Figure 4:**
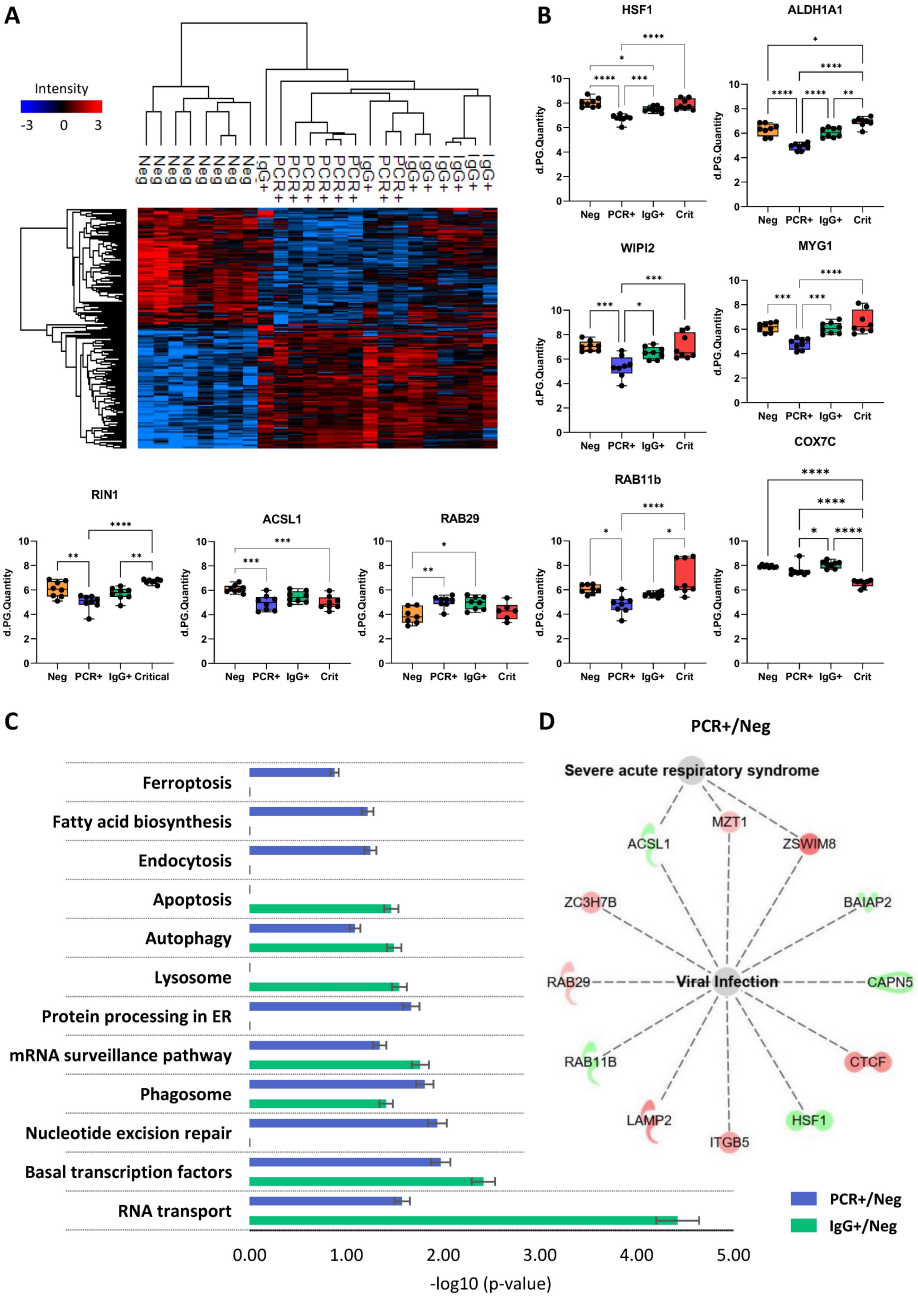
** Proteomic changes in ECFCs exposed to asymptomatic serums. A)** Hierarchical cluster representing the differential protein profiles for ECFCs incubated with the serum of asymptomatic (PCR+, IgG+) and COVID-19 Neg donors. **B)** Graphical representation of the LFQ intensities registered for proteins altered in ECFCs exposed to asymptomatic serums. **C)** Altered pathways in ECFCs stimulated with serum from asymptomatic donors compared to ECFCs+Neg cells. The p-values (-log10) obtained by Kegg Pathway platform and the calculated SEM are represented. **D)** IPA functional network with proteins up- (red) or down-regulated (green) in PCR+ *vs* Neg correlated with viral infection and severe acute respiratory syndrome. *p-value<0.05, **p-value<0.01, ***p-value<0.001, ****p-value<0.0001.

Focusing on the response of ECFCs to asymptomatic serums, our results indicated that the expression patterns of ECFCs+PCR or ECFCs+IgG cells were similar, compared with ECFCs exposed to the serum of COVID-19 negative donors (Figure 4A). Some of these changes are shown in Figure 4B.

According to KEGG pathways, proteins altered in ECFCs+PCR or ECFCs+IgG appeared to participate, among others, in mRNA surveillance (SSU72, PELO) and RNA transport (RPP40, GEMIN7, SNUPN), and also related to phagosomes (ITGB5, SEC61G, LAMP2) or autophagy (CTSL, LAMP2, WIPI2) (Figure 4C). Similarly, the Ingenuity Pathway Analysis (IPA) classification platform connected several proteins up- and downregulated in response to the asymptomatic serum factors to viral infection, apoptosis and autophagy (Table S6) and, in the case of the ECFCs+PCR group, protein changes were specifically associated with severe acute respiratory syndrome (ACSL1, MZT1, ZSWIM8) (Figure 4D).

These data corroborate our initial findings suggesting that SARS-CoV-2 promotes molecular alterations even in total or partial absence of classical symptoms 7, and that these changes persist when the infection has disappeared (PCR-/IgG+).

### Proteomic changes in ECFCs in response to the serum of critical patients

**Figure 5:**
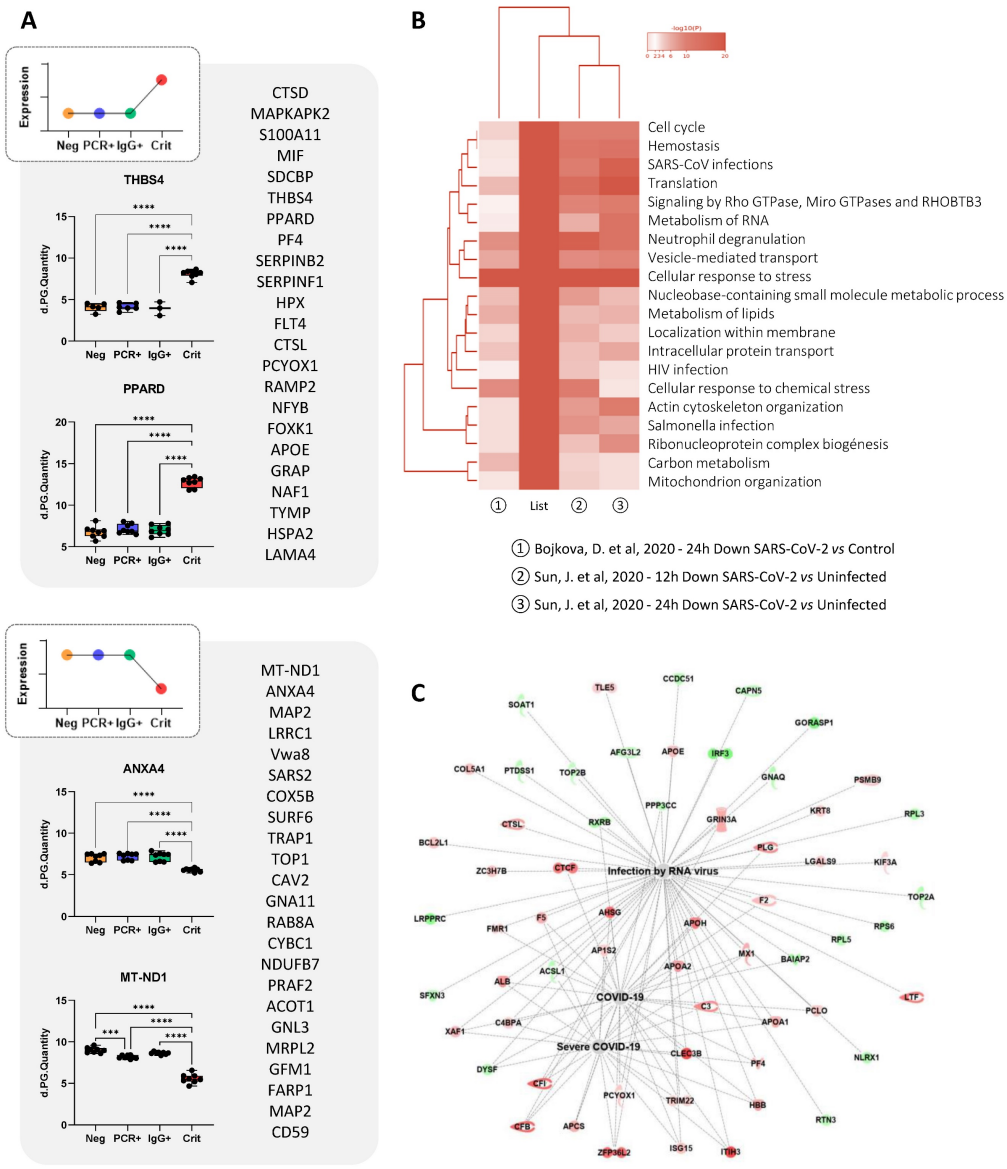
** ECFCs differential protein expression in response to the serums of critical patients. A)** Schematic representation of proteins altered (up or down-regulated) in ECFCs only after incubation with the with serums from critical patients. Also, representative graphs with the LFQ intensities registered for some of the altered proteins are shown. **B)** Hierarchical functional clustering provided by by Coronascape for proteins differentially expressed in ECFCs in response to critical COVID-19 serums (*vs* Neg). See Figure S1 for full information.** C)** IPA functional network including proteins up- (red) or down-regulated (green) in ECFCs+Crit *vs* ECFCs+Neg correlated with RNA virus and COVID-19 infection.

As indicated above, most changes were seen in ECFCs in response to the serum of COVID-19 critical patients. For example, proteins like S100A11, PF4, PPARD, MIF, THBS4, or ITGB5, were up-regulated in ECFCs+Crit compared to the other groups (Figure 5A). Based on the results provided by “coronascape” platform 32, supported itself by several database online platforms such as Reactome, Go Biological or KEGG pathways, the proteins differentially expressed in ECFCs+Crit were involved in many different pathways associated to viral infection, including RNA metabolism, ribonucleoprotein complex biogenesis, signaling by RhoGTPAses, or vesicle mediated transport, as well as SARS-CoV and human immunodeficiency virus (HIV) infections, neutrophil degranulation or cellular response to stress, among others (Figure 5B and Figure S1). More directly, IPA platform correlated the protein changes detected in ECFCs (Crit *vs* Neg) with RNA virus and COVID-19 infection (Figure 5C), as well as with functions altered or up-regulated in severe COVID-19 patients, such as immune response, coagulation, infection and apoptosis (Table 1).

### Discriminating proteins highlighted by machine learning prediction tools

A multinomial logistic regression (MLR), Näive Bayes (NB) and linear support vector machines (LSVM) classifiers were trained and cross-validated to automatically classify ECFCs+Neg, ECFCs+PCR, ECFCs+IgG and ECFCs+Crit samples. The application of these three machine learning techniques reported several proteins highly discriminating between the four groups. The performance of all classifiers was very promising, as evidenced by the estimated metrics. ALDH1A and MT-ND1 molecules were selected as relevant for the three predictive models while SDCBP, HTRA2, CAPN5, STX10, and RIN1 proteins appeared as features with high predictive value in two of the validated models (Table 2 and Figure S2).

### Cardiovascular related proteomic changes in ECFCs treated with the serum of infected individuals

**Figure 6:**
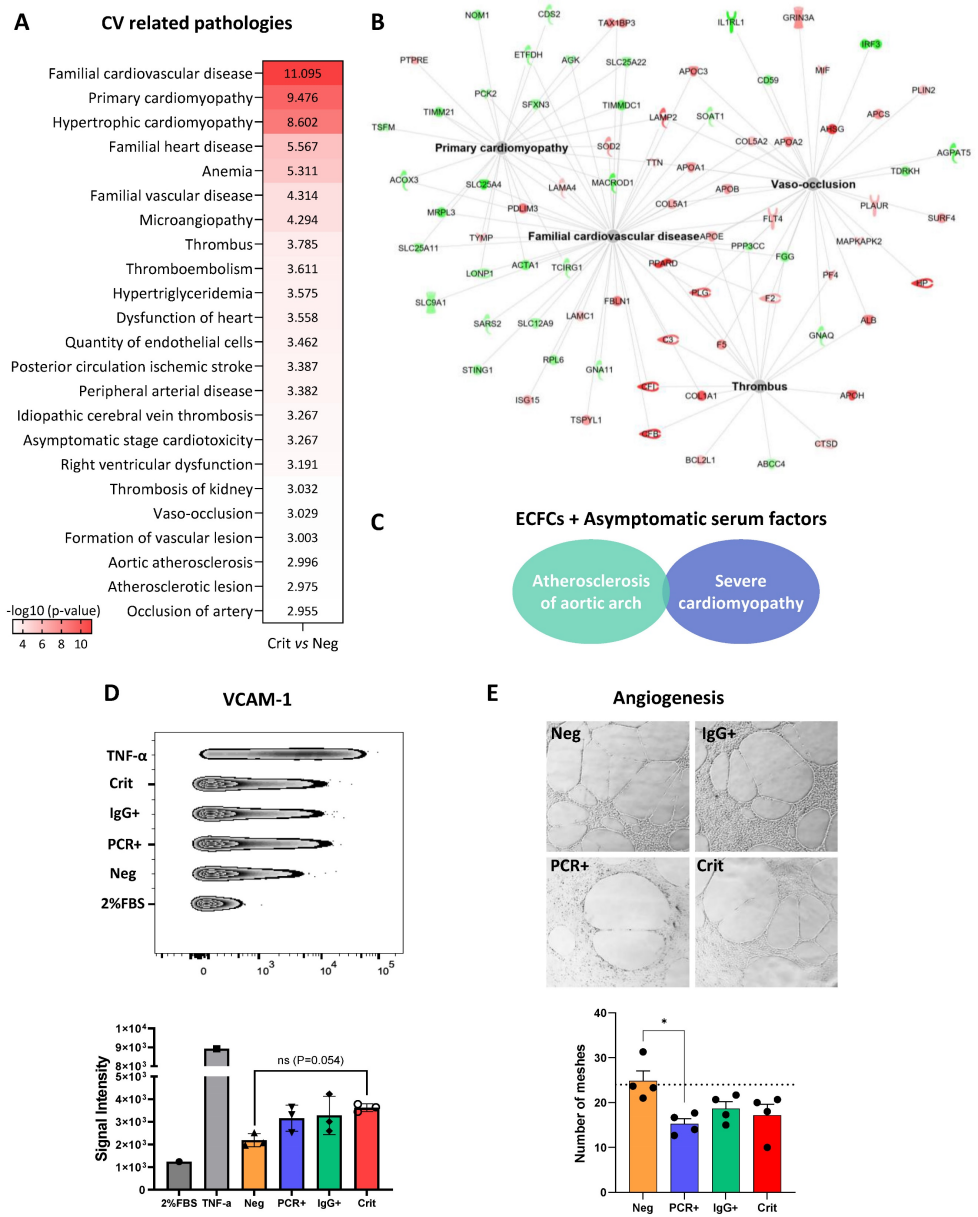
** Proteomic changes in ECFCs related with cardiovascular-related pathologies. A)** Altered functions and diseases related with CVDs in ECFCs incubated with the serum from critical patients (Crit *vs* Neg), provided by IPA.** B)** Functional network of CV related pathologies (Crit *vs* Neg), with proteins up- (red) or down-regulated (green). **C)** Altered diseases related with CVDs in ECFCs incubated with serum from asymptomatics (PCR+ and IgG+), compared with ECFCs+Neg samples. D) VCAM-1 levels increased in ECFCs in response to the serums from asymptomatic and critical donors compared with ECFCs+Neg samples. E) Representative images of the reticular structures formed by ECFCs after 24h incubation with the serum of Negative, asymptomatics and critical patients. Differences between ECFC+PCR and ECFC+Neg were statistically significant (*p < 0.05).

Many of the protein changes seen in ECFCs incubated with the serum from critical patients (ECFCs+Crit) were associated with cardiovascular-related pathologies, including vaso-occlusion, atherosclerosis and thrombotic related processes, cardiomyopathy, ischemic stroke or peripheral artery disease among others (Figure 6A-B and Table S7). Among these, proteins like lysosome-associated membrane protein 2 (LAMP2) and surfeit locus protein 4 (SURF4) were over-expressed in ECFCs+Crit cells but also in ECFCs treated with sera from asymptomatics (ECFCs+PCR and ECFCs+IgG). Indeed, although to a much lesser extent, asymptomatic sera also promoted changes associated with CVDs such as atherosclerosis of aortic arch and severe cardiomyopathy (Figure 6C). In agreement with the proteomic results, functional assays reported an up-regulation of the vascular cell adhesion molecule (VCAM1), a marker of endothelial dysfunction and inflammation correlated with CVD 33, in response to all positive COVID-19 serums, whether from asymptomatics or critical patients (Figure 6D). Moreover, the angiogenic potential of ECFCs was impaired (Figure 6E).

### Interaction networks between COVID-19 serum and ECFCs altered proteins

**Figure 7:**
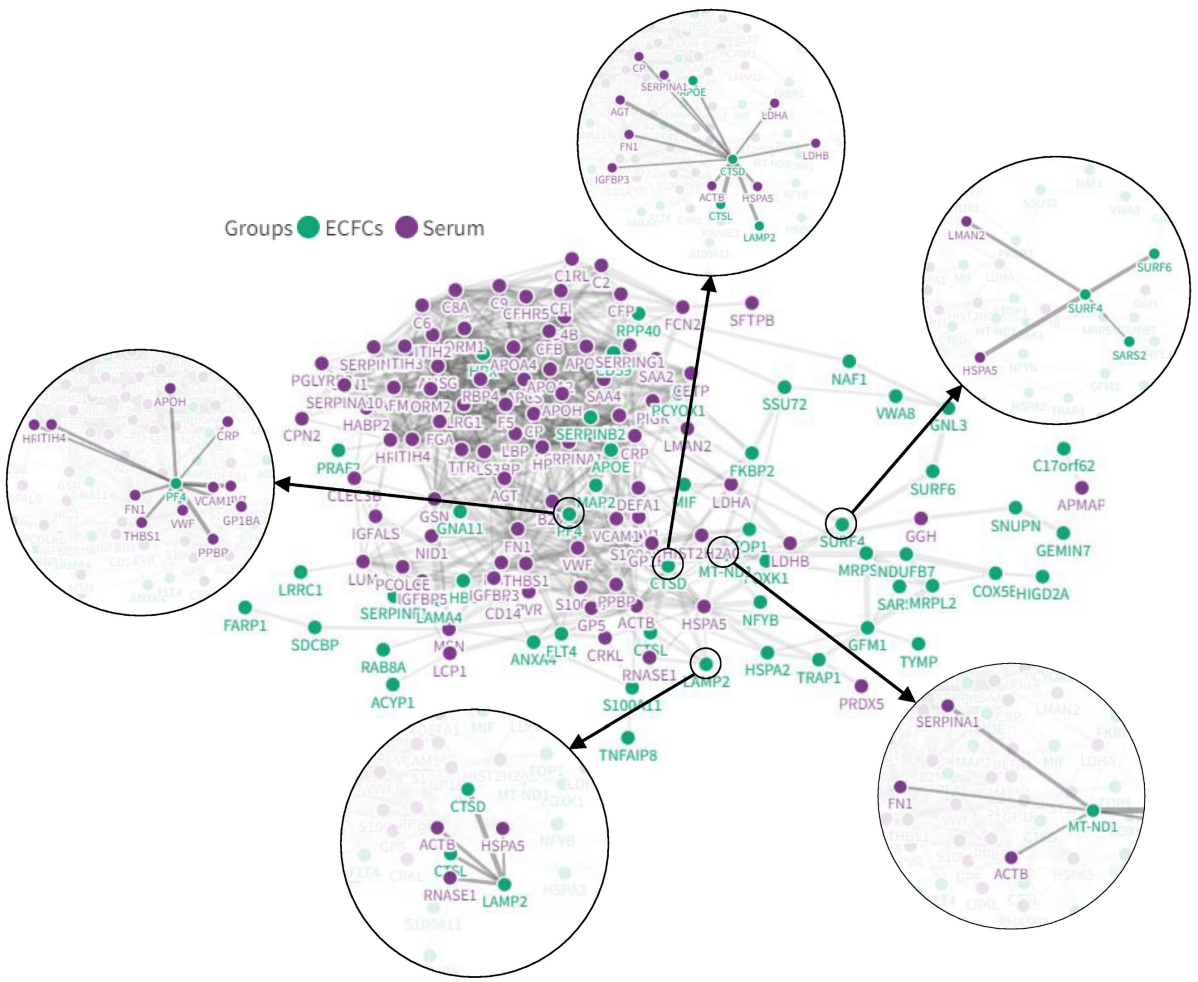
***In-silico* analysis with the potential interactions between altered proteins in the COVID-19 patients' serum and ECFCs.** Some of the most relevant protein interactions connected to CVD have been highlighted.

Different serum proteins that have been reported as altered after COVID-19 infection (i.e. CRP, von Willebrand factor, HSPA5, or LMAN2) in several proteomic studies (Table S9) were directly connected with the protein changes seen in ECFCs in the current approach in response to COVID-19 positive sera (SURF4, LAMP2, PF4, CTSL or CTSD, among others) (Figure 7).

## Discussion

At present, different serum markers associated to the severity of SARS-CoV-2 have been disclosed, including the C-reactive protein (CRP), procalcitonin (PCT), or ferritin 34, as well as the so-called “cytokine storm” (IL-6, TNF-α and other pro-inflammatory factors) 35, or markers directly related to coagulation (D-dimer) or cardiac injury, such as troponin, N-terminal (NT) proB-type natriuretic peptide (BNP), or creatine kinase (CK) 36, 37. Additionally, serum miRNA targeting ACE2 or other genes have also been reported 38, 39. Many of these biomarkers are indicators of tissue injury or even the severity of the disease, but they cannot explain the differential predisposition of individuals to respond against the infection, or why many individuals suffer from long-term sequelae, with no clear explanation yet of how COVID-19 symptoms remain. Therefore, it is still a long way to understand the mechanisms of action of SARS-CoV-2 as well as the organism´s response to this virus.

Endothelial dysfunction represents one of the most characteristic effects of SARS-CoV-2 and a major underlying mechanism responsible of CV complications in COVID-19 patients 40. Indeed, critical patients present elevated levels of endothelial biomarkers compared to non-critical ones, suggesting a prognostic role of endothelial dysfunction in COVID-19 disease 41. Furthermore, the presence of increased levels of ECFCs in 3 months post-COVID-19 patients compared to healthy subjects, might also represent a marker of post-COVID endothelial damage 9. Of note, ECFCs are negatively affected by adverse environments, and altered levels and or functions of ECFCs have been linked to CV events 17, 22, 42, 43.

Remarkably, our results indicate that the incubation of ECFCs with the serum of COVID-19 positive individuals, asymptomatic (PCR+ or IgG+) or critical patients, promotes changes at the protein level that resemble alterations associated endothelial dysfunction and viral infection. Indeed, different proteins associated to viral infection like the Long-chain Acyl-CoA synthetase 1 (ACSL1), Calpain-5 (CAPN5) or Syntaxin 10 (STX10), appeared down-regulated in ECFCs after stimulation with the serum of COVID-19 positive individuals. The last two, CAPN5 and STX10, were identified by predictive tools as highly discriminating proteins between ECFCs treated with “infected” serums and ECFCs incubated with COVID-19 negative serums (ECFCs+Neg). Noteworthy, ACSL1 has been proposed as an antiviral agent, by enhancing the production of interferon I (IFN-I) and mediating apoptosis through the PI3K/Akt signaling pathway in response to the avian leukosis virus 44. ACSL1 down-regulation might reflect a viral strategy to reduce IFN-I levels, in agreement with previous studies demonstrating the potential of SARS-CoV-2 to prevent IFN-I signaling, and also to down-regulate the sensitivity and response of SARS-CoV-2 to IFN-I 45, 46. On the other hand, CPN5 down-regulation might represent a protective mechanism in ECFCs, given the role that CPN5 and other calpain proteins seem to play in viral entry and replication 47-49. Indeed, several calpain inhibitors such as MG132, II and XII, appear to inhibit SARS-CoV and SARS-CoV-2 at the early stages of viral replication 50, 51. Finally STX10**,** a protein that facilitates vesicle´s fusion during intracellular trafficking of proteins and other cellular components 52, has been found to interact with the SARS-CoV-2 accessory proteins ORF3 and ORF7b, supporting its own replication and survival 46. Thus, the modulation of STX10 or similar trafficking proteins might represent an alternative therapeutic target against this virus.

We also found protein changes only in ECFCs treated with the serum of asymptomatic individuals (PCR+ or IgG+), in agreement with previous results 7. These changes correlated, among others, with mRNA surveillance or RNA transport, phagosomes and autophagy. Different studies have evaluated the potential role of autophagy into viral invasion and replication 53, 54. Although autophagy represents a protective strategy from the host cells to eliminate the intruding viruses 55, viruses can interfere or evade the autophagic process, and even use the autophagic machinery for their own replication 56. Recent reports indicated that SARS-CoV-2 efficiently avoids the anti-viral functions of autophagy, regulating autophagy by interaction of its factors ORF3 and NSP6 with cell host autophagic factors such as WIPI2 or LAMP2 proteins 57, two proteins altered in response to the serums from COVID-19 patients.

Remarkably, the serum of critical patients promoted the highest number of protein changes in ECFCs, with a protein profile clearly differing from the rest of the conditions analyzed. Among them, Galectins-8 and 9 (LGALS8 and LGALS9) appeared up-regulated. LGALS9 has been associated to the severity of HIV viral infection 58, 59, and its expression increases in response to many different viruses, including HIV, HCV, hepatitis B virus, herpes simplex virus, influenza virus, or dengue virus 60. Similarly, LGALS8 appears to recognize the SARS-CoV-2 protein spike S1, highly glycosylated, which activates an antiviral autophagy mechanism, which SARS-CoV-2 counteracts by cleavage of LGALS8 61. Therefore, LGALS8 up-regulation might represent a protective mechanism against the virus that is triggered only under severe conditions (ECFCs+Crit). Further studies are needed to validate this hypothesis.

Like LGALS8, Polypyrimidine tract binding protein (PTBP1) and two members of the lysosomal cysteine protease family, Cathepsin L (CTSL) and D (CTSD), were also highly upregulated in ECFCs+Crit cells. PTBP1 binds mRNA and is essential for viral translation and replication 62. Recent studies suggest that PTBP1 cleavage or inhibition might promote an inhibitory effect on SARS-CoV-2 replication 61. Similarly, CTSL, a matrix-degrading enzyme upregulated in chronic inflammation 63, is used by SARS-CoV and SARS-CoV-2 viruses to cleave and activate the spike protein S between the residues Thr696 and Met697 in the S1-S2 domains, promoting the S-protein mediated cell-cell fusion and the release of the virus´s genome into the host cell^64^, ^65^, Indeed, several CTSL inhibitors have shown promising results by impairing the entry of the virus and further replication 64, 65.

Several proteins associated with mitochondrial dysfunction, such as Cytochrome c oxidase subunit 7C (COX7C) and NADH-ubiquinone oxidoreductase chain 1 (MT-ND1), were also highlighted by machine learning algorithms. Both proteins were downregulated in ECFCs+PCR and more significantly in ECFCs+Crit. Notably, like many other viruses, SARS-CoV-2 is thought to modulate mitochondrial dynamics in its own benefit 66-68, by sending its genetic material towards the mitochondria to influence ROS production, mitophagy, iron storage, platelet coagulability, and cytokine production stimulation, supporting viral replication 69, 70. Thus, the infection of endothelial cells by SARS-COV-2 might contribute to mitochondrial dysfunction and oxidative stress, key players on the initiation of chronic inflammation and endothelial damage 71. Drugs targeting mitochondria and/or some of the proteins highlighted here could be considered as potential tools for protecting the endothelium in severe forms of COVID-19 72.

Amidst all, many protein changes in ECFCs+Crit cells were linked to CVDs, including cardiomyopathy (also seen in ECFCs+PCR cells), ventricular dysfunction, vaso-occlusion and thrombosis, ischemic stroke or even kidney thrombosis. In agreement with these results, functional assays reported that the levels of VCAM1 were up-regulated in response to all positive serums, as already seen in COVID-19 patients 73-75, while the angiogenic potential of ECFCs was impaired. Both situations are indicative of endothelial dysfunction associated with cardiovascular events 33, 75.

Some of the CVDs-related proteins identified were connected, by *in silico* analysis, to several proteins that have been found altered in the serum of critical patients (Figure 7). For example, the Platelet factor 4 (PF4), a protein highly up-regulated only in ECFCs+Crit cells, was connected to PPBP, THBS1, FN1, CRP or vWF. Some of these proteins, including PF4, participate in the coagulation pathway, which becomes highly activated in severe COVID-19 patients, being responsible of thrombotic events 76, 77. For instance, PF4 interaction with heparin promotes platelet aggregation and thrombi formation. Moreover, the formation of PF4-vWF complexes might propagate the risk of thrombosis in an heparin dependent manner 78. PF4 is also highly secreted in response to viral infection, contributing to neutrophils recruitment, among others 79. Finally, high levels of anti-PF4/heparin antibodies have been found in hospitalized patients, although this seem to be associated with the severity of COVID-19 rather than a marker of thrombotic risk 80. Thus, up-regulation of serum markers such as vWF or CRP might be directly or indirectly associated with the upregulation of PF4 or other thrombotic markers in ECFCs.

LAMP2 or SURF4 were also up-regulated in ECFCs in response to the serum of critical patients as well as in ECFCs with PCR+ serum (at the highest peak of infection) and, interestingly, in individuals that had overcome the infection without apparent symptoms (PCR-/IgG+). These proteins were connected *in silico* to several serum markers upregulated in critical COVID-19 patients, including the chaperone Heat shock protein HSPA5, also called glucose regulating protein 78 (GRP78), a glycoprotein upregulated as result of ER stress mediated by COVID-19 infection 81. Regarding LAMP2, this protein has been associated to autophagy, more precisely, chaperone mediated autophagy (CMA), activated against oxygen and glucose deprivation, conferring cardiomyocyte resistance against such stress *in vitro* 82. Moreover, LAMP2 deficiency and disrupted autophagy are responsible of Danon disease, a rare cardiomyopathy that usually leads to profound hypertrophic cardiomyopathy resulting in death or requiring transplantation in men 83, 84. On the other hand, nothing has been described, to our knowledge, associating directly LAMP2 with SARS-CoV-2 infection. Given the implication of LAMP2 in autophagy and the effect that impaired autophagy has over the CV system, future studies should evaluate the potential modulation of this virus over LAMP2 as well as other autophagic proteins as an alternative to block or avoid the progression towards more complicated and long-term vascular situations.

Finally, the cargo receptor SURF4 is an integral ER membrane protein involved in the assembly and packaging of proteins into ER‐derived transport vesicles. This protein regulates, among others, insulin 85 or PKSL9^86^ secretion via ER export. Since both proteins are important regulators of both glucose and plasma cholesterol levels, up-regulation of SURF4 in ECFCs+Crit might reflect, again, the capacity of SARS-CoV-2 to alter lipid and glucose metabolism 87 in its own benefit.

## Conclusion

Overall, the incubation of adult ECFCs with the serum factors of SARS-CoV-2 infected individuals constitutes an optimal approach to evaluate the endothelial cells response to SARS-CoV-2 depending on the severity of COVID-19 disease, in agreement with previous results 7. Indeed, machine learning algorithms have reported some specific proteins such as STX10, SDCBP, CAPN5, MT-ND1 or ALDH1A1 as highly discriminating proteins between the groups compared. Many proteins identified here have been associated to viral invasion, extravasation and replication, while many others provide insights of the potential mechanisms of the virus to alter the cell host machinery in its own benefit (autophagy, mitochondrial dysfunction, etc). Moreover, the serum factors of infected individuals compromised the angiogenic potential of ECFCs, while promoted changes in the endothelial cells resembling cardiovascular-related pathologies. These changes might as well explain the activation of long-term vascular sequelae after SARS-CoV-2 infection.

While future research is required to determine which factors might indeed be promoting the protein changes seen here, as well as to further validate the involvement of the proteins identified, some of the proteins highlighted might be taken as potential candidates for therapeutic approaches to neutralize SARS-CoV-2 effect over the endothelium and hopefully to prevent potential CV events in COVID patients and post-COVID individuals.

## Supplementary Material

Supplementary tables.

## Figures and Tables

**Table 1:** ** Functional classification of differentially expressed proteins in ECFCs incubated with critical COVID-19 patient's serum (Crit) *vs* incubated with healthy donor's serum (Neg).** Protein classification was made with the IPA software based on biomedical literature and integrated databases. The table shows the most probable functions in which the proteins of interest are involved, p-value, activation z-score, gene names and number

Functions	P-value	Z-score	Gene names	Number of IDs
Immune response of leukocytes	9.95E-04	2.441	APCS, APOA1, APOA2, APOE, BCL2L1, C3, CD59, HSDL1, IGHG3, IGHM, IL1RL1, IRF3, ISG15, LGALS9, LTF, MAPKAPK2, MIF, MRTFA, PF4, PLAUR, PLCG2, SOAT1, TCIRG1	23
Degranulation	1.23E-08	1.672	A2M, ABCC4, ACAA1, ADAM10, AHSG, ALB, APOA1, APOH, APOOL, C3, CD59, CLEC3B, CMTM6, CTSD, CYB5R1, DHCR7, F2, F5, FERMT3, FGG, FTH1, HBB, HP, ITGAE, ITIH3, KRT1, LAMP2, LGALS9, LTF, MAGT1, METTL7A, MIF, NCSTN, NDUFC2, ORMDL3, PF4, PLAUR, PLCG2, PLG, PPP3CC, PSMB7, RICTOR, S100A11, STING1, SURF4, TCIRG1, TMEM179B, TTN	48
Coagulation	3.46E-04	0.751	A2M, APOE, APOH, BLOC1S6, C3, CD59, COL1A1, EHD3, F2, F5, FGG, GNA11, GNAQ, HBB, HP, MRTFA, PF4, PLAUR, PLCG2, PLG, SERPINF1	21
Viral Infection	1.13E-05	0.376	ACSL1, ADAM10, AFG3L2, AHSG, ALB, AP1S1, AP1S2, APCS, APOA1, APOA2, APOB, APOBEC3F, APOE, APOH, BAIAP2, BCL2L1, C3, C4BPA, CAPN5, CCDC51, CFB, CFI, CLEC3B, COL5A1, CTCF, CTSL, DYSF, ELOVL5, F2, F5, FMR1, GCAT, GNAQ, GORASP1, GPAT3, GRIN3A, HBB, HP, IGHM, IRF3, ISG15, ITGB5, ITIH3, KIF3A, KRT8, LAMP2, LGALS9, LONP1, LRPPRC, LTF, MAGT1, MARCHF2, MCL1, MIF, MTX1, MX1, MZT1, NLRX1, PARP12, PCLO, PCYOX1, PF4, PLCG2, PLG, PPAN, PPP3CC, PSMB9, PTDSS1, RICTOR, RPL3, RPL38, RPL5, RPS6, RTN3, RXRB, SFXN3, SOAT1, STING1, TCIRG1, TLE5, TOP2A, TOP2B, TRIM22, XAF1, ZC3H7B, ZFP36L2, ZSWIM8	87
Cell Infection	8.30E-04	0.225	AFG3L2, APCS, APOE, APOH, C3, CAPN5, CCDC51, CFI, COL5A1, CTSL, F2, GNAQ, GORASP1, IRF3, KIF3A, LGALS9, LRPPRC, LTF, MX1, NLRX1, PTDSS1, RPL3, RPL5, RPS6, RTN3, SFXN3, TLE5, ZC3H7B	28
ROS Generation	2.14E-05	-0.612	AATF, ALB, ALDH2, APOA1, APOE, BCL2L1, BNIP3, DHCR24, F2, HP, ITM2B, LTF, MACROH2A1, MIF, PLAUR, PPARD, SERPINF1, SOD2, TXNRD1	19
Necrosis/Apoptosis	1.98E-05	-1.181	A2M, AATF, ABCC4, ABCG2, ACP1, ACSL1, ADAM10, ALB, ALDH2, APOA1, APOB, APOC3, APOE, ARMC10, BCL2L1, BNIP3, C3, CD59, CDCA2, CFB, CHP1, COL1A1, CS, CTCF, CTSD, DAP3, DCK, DHCR24, DHCR7, DYSF, EBNA1BP2, ELOVL5, F2, F5, FAIM, FAP, FECH, FLT4, FMR1, FTH1, GLS, GLUD1, GNAQ, GNL3, GPNMB, GRIN3A, HADHA, HBB, HTRA2, IARS2, IDH2, IGHM, IL1RL1, IMMT, IRF3, ISG15, ITM2B, KIF3A, KRT8, LAMA4, LAMA5, LAMP2, LGALS9, LONP1, LRRC8A, LTF, MAPK7, MAPKAPK2, MCL1, MIF, MRTFA, MST1, MTCH2, MX1, MYOC, NCSTN, NDUFA13, NLRX1, ORMDL3, PAK4, PCK2, PEX11B, PF4, PLAUR, PLCG2, PLG, PMVK, POLR2H, PPARD, PPP1R11, PTPRE, PTPRF, PTRH2, RBM3, RFK, RGN, RICTOR, RNASEH2A, RPL27A, RPL3, RPL38, RPL5, RPL6, RPS24, RPS6, RRP1B, S100A11, SEC61G, SERPINB2, SERPINF1, SLC16A1, SLC1A1, SLC25A11, SLC25A4, SLC9A1, SOAT1, SOD2, SRPX, STING1, SURF1, TAF4, THBS4, TNFAIP8, TNFAIP8L1, TOP1, TOP2A, TOP2B, TRAP1, TTN, TXNRD1, TYMP, VTI1A, XAF1, ZMPSTE24	134
Autophagy	8.32E-04	-1.306	ABHD5, ACSL1, BAIAP2, BCL2L1, BNIP3, CTSD, CTSL, ERCC4, FOXK1, IRF3, LAMP2, LAMTOR4, LETM1, MCL1, NAF1, NLRX1, ORMDL3, PLG, RAB8A, RICTOR, SLC25A4, SLC9A1, SOAT1, SOD2, SRPX, STING1, TCIRG1, TOMM22, TOMM6, TRIM22, VTI1A, ZMPSTE24	32
Mitochondrial Dysfunction	1,81E+01		ACO2, ATP5ME, ATP5MF, ATP5MG, ATP5PB, ATP5PD, ATPAF2, COX4I1, COX5B, COX6C, COX7B, COX7C, CPT1A, HTRA2, MT-CO3, MT-ND1, MT-ND5, NCSTN, NDUFA13, NDUFA9, NDUFB11, NDUFB3, NDUFB5, NDUFB6, NDUFB7, NDUFB8, NDUFS2, RHOT2, SOD2, SURF1, UQCR10	31

**Table 2:** ** Machine learning models.** Performance of validated machine learning models to discriminate between ECFCs treated with either the serum from: a) critical patients; b) from asymptomatic PCR+ donors samples; c) from asymptomatic IgG + donors samples; and d) from COVID negative donors

Model	Acc	AUC	TP-FP	Recall	Kappa	Gene names
MLR	1.00	1.00	1.00-0.00	1.00	1.00	SDCBP, CAPN5, HTRA2, STX10, ALDH1A1, MT-ND1, RIN1
NB	0.88	0.95	0.88-0.04	0.88	0.83	C5ORF51, MCRIP1, CCS, EIF3H, RPP40, CD2BP2, ALDH1A1, MT-ND1, COX7C, HNRNPUL2, LSM12, CCNYL1
LSVM	0.97	0.98	0.97-0.01	0.97	0.96	DDX39A, SDCBP, CAPN5, HTRA2, STX10, ALDH1A1, APOH, MT-ND1, RIN1

*Acc:* accuracy, *AUC:* area under the receiver operating characteristic curve, *TP:* true positive, *FP:* false, *MLR:* multinomial logistic regression, *NB:* Naïve Bayes, *LSVM:* linear support vector machines.

## Abbreviations

AUC): Area under the ROC curve
CRP): C-reactive protein
CAPN5): Calpain-5
CV): Cardiovascular
CVDs): Cardiovascular diseases
CTSL): Cathepsin L
CTSD): Cathepsin D
CACs): Circulating angiogenic cells
CECs): Circulating endothelial cells
CK): Creatine kinase
COX7C): Cytochrome C oxidase subunit 7C
DENV): Dengue virus
ECFCs): Endothelial colony forming cells
LGALS8): Galectin-8
LGALS9): Galectin-9
HBV): Hepatitis B virus
HSV): Herpes simplex virus
HIV): Human immunodeficiency virus
IFN-I): Interferon I
LFQ): Label free quantitative
LSVM): Linear support vector machines
ACSL1): Long-chain Acyl-CoA synthetase 1
LAMP2): Lysosome-associated membrane protein 2
MS): Mass spectrometry
MLR): Multinomial logistic regression
NT): N-terminal
MT-ND1): NADH-ubiquinone oxidoreductase chain 1
NB): Näive Bayes
PF4): Platelet factor 4
PTBP1): Polypyrimidine tract binding protein
PCA): Principal component analysis
BNP): proB-type natriuretic peptide
PCT): procalcitonin
SARS-CoV-2): Severe acute respiratory syndrome coronavirus 2
SURF4): Surfeit locus protein 4
STX10): Syntaxin 10
